# Psychiatric symptoms and risk factors in adults born preterm with very low birthweight or born small for gestational age at term

**DOI:** 10.1186/s12888-019-2202-8

**Published:** 2019-07-17

**Authors:** Astrid M. W. Lærum, Solveig Klæbo Reitan, Kari Anne I. Evensen, Stian Lydersen, Ann-Mari Brubakk, Jon Skranes, Marit S. Indredavik

**Affiliations:** 1 Department of Clinical and Molecular Medicine, Faculty of Medicine and Health Sciences, Norwegian University of Science and Technology, Unit for Pediatrics, 6th floor, Kvinne-barn-senteret, Olav Kyrres gt. 11, 7030 Trondheim, Norway; 2 Children’s Clinic, St. Olavs Hospital, Trondheim University Hospital, Trondheim, Norway; 3 Department of Mental Health, Faculty of Medicine and Health Sciences, Norwegian University of Science and Technology, Trondheim, Norway; 4 Department of Mental Health, St. Olavs Hospital, Trondheim University Hospital, Trondheim, Norway; 5 Department of Public Health and Nursing, Faculty of Medicine and Health Sciences, Norwegian University of Science and Technology, Trondheim, Norway; 6 Unit for Physiotherapy Services, Trondheim Municipality, Norway; 7 Department of Physiotherapy, Faculty of Health Sciences, Oslo Metropolitan University, Oslo, Norway; 8 Department of Pediatrics, Sørlandet Hospital, Arendal, Norway

**Keywords:** Low birthweight, Mental health, Psychiatric symptoms, Risk factors, Autism

## Abstract

**Background:**

We aimed to examine psychiatric symptoms in adults born preterm with very low birthweight or born at term small for gestational age compared with normal birthweight peers, and examine associations with perinatal factors and childhood motor and cognitive function.

**Methods:**

In this longitudinal cohort study, one preterm born group with very low birthweight (VLBW: birthweight ≤1500 g), one term-born Small for Gestational Age (SGA: birthweight <10th percentile) group and one term-born non-SGA control group, were assessed at 26 years of age. Primary outcomes were scores on self-reported questionnaires: Achenbach System of Empirically Based Assessment - Adult Self-Report, The Autism-Spectrum Quotient and Peters et al. Delusions Inventory. Exposure variables were perinatal data, while childhood motor and cognitive function were examined as possible early markers.

**Results:**

Both the preterm VLBW and the term SGA group reported higher levels of attention, internalizing and externalizing problems compared to the control group. In addition, the VLBW participants reported more critical items and a higher proportion had intermediate level autistic traits, while the SGA participants reported more intrusive behavior. Increasing length of respiratory support and hospital stay in the neonatal period, and motor problems in early adolescence, were associated with adult psychiatric symptoms in the VLBW group.

**Conclusions:**

Psychiatric symptoms were frequent in the preterm VLBW group and also in the term-born SGA group. Those who were sickest as babies were most at risk. Motor problems can possibly serve as an early marker of adult psychiatric symptoms in low birthweight individuals.

**Electronic supplementary material:**

The online version of this article (10.1186/s12888-019-2202-8) contains supplementary material, which is available to authorized users.

## Background

Preterm birth and low birthweight are risk factors for later neurodevelopmental difficulties, including psychiatric symptoms, motor problems and poor cognitive and adaptive/social functioning [1–4]. The most common psychiatric symptoms involve anxiety and attention deficit, but traits of autism spectrum disorder (ASD) and symptoms of depression and schizophrenia are also reported in adulthood [5–8]. Motor problems in preterm/very low birthweight (VLBW: ≤1500 g) individuals are most often identified in childhood, and range from minor difficulties to more severe outcomes, like cerebral palsy (CP) [2, 9]. Cognitive function is reportedly below that of their term-born and normal birthweight peers, but its relation to psychiatric morbidity remains unclear [10]. Term-born small for gestational age (SGA) children are also reported to have reduced neurodevelopmental function [11] compared with their normal birthweight peers, with lower fine motor [12] and cognitive function [13], and more emotional, behavioral and attention deficit symptoms in adolescence [14].

To guide early intervention and limit unfavorable neurodevelopmental outcomes in VLBW individuals, attempts have been made to identify predisposing pre- and perinatal factors. Intrauterine growth retardation, maternal smoking and substance use, low birthweight, low gestational age, birth asphyxia, neonatal respiratory distress, perinatal infections and intraventricular hemorrhage (IVH) are some of the proposed medical risk factors [15, 16]. Childhood motor problems have been associated with reduced cognitive abilities, social skills and language development [17, 18], but the relation to adult psychiatric problems is less studied. Hence, further knowledge about adult psychiatric outcomes and possible predictive factors is needed.

This study aimed to assess self-reported psychiatric symptoms in adults born either preterm with VLBW or at term SGA, compared to a term-born control group with normal birthweight. We also aimed to investigate possible perinatal risk factors and early neurodevelopmental markers of unfavorable mental health outcome.

We hypothesized that the preterm born VLBW and term-born SGA group would have a higher overall self-reported psychiatric symptom load, and specifically more symptoms of anxiety and depression, attention problems, autism spectrum traits and minor psychotic manifestations/odd beliefs, compared to the control group. We anticipated that perinatal risk factors, and reduced childhood motor and cognitive function would be associated with more psychiatric symptoms in adulthood in the VLBW and the SGA group.

## Methods

### Study design

**Fig. 1:**
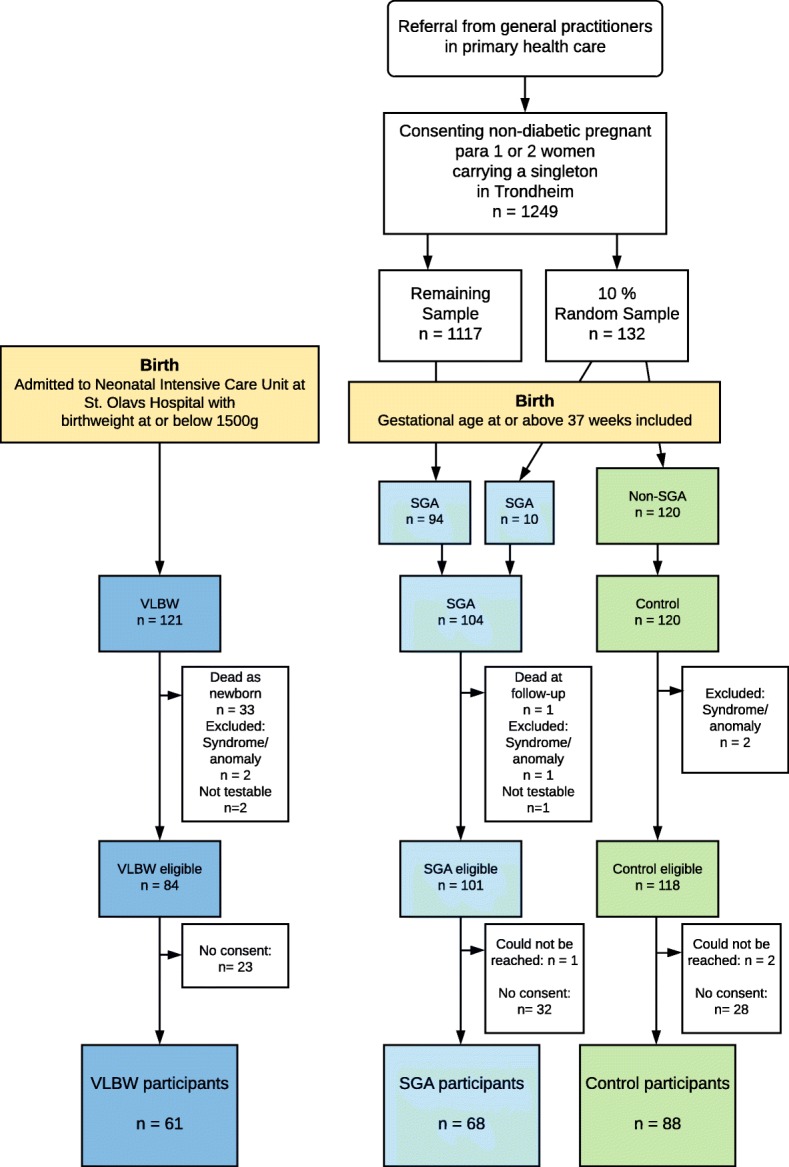
Flowchart of participation in the three study groups

As part of a prospective cohort study, a preterm born VLBW group, a term-born SGA group and a term-born non-SGA control group were examined at age 26 years. All participants were Caucasian and born between 1986 and 1988. A flow chart of participation is shown in Fig. 1. The preterm VLBW subjects had all been admitted to the Neonatal Intensive Care Unit (NICU) at the St. Olavs University Hospital in Trondheim. They were either born at this hospital (geographical cohort) or referred from a local hospital in the region of Central Norway, thus constituting an extended sample, which was used in this study. The term-born SGA and control participants were born to women residing in the Trondheim area, and recruited as part of a multicenter study [19]. At enrollment, a 10% sample of these women were randomly selected for participation using a closed envelope procedure. At birth, all children born term non-SGA in this random sample were included as controls for prospective follow-up. Children born term SGA in this random sample, and all children born term SGA in the remaining study population were included in the SGA group. At follow-up at age 1 and 5 years, only children born in 1988 were included from the VLBW group. At follow-up at 14, 19 and 26 years, participants born 1986 to 1988 were included. All questionnaires used at the 26-year assessment were authorized Norwegian translations and were completed by the participants during project attendance. An experienced clinician, blinded to group adherence and previous results, was present to clarify any question. For a few participants the questionnaires were sent and received by mail. The prevalence of psychiatric diagnoses at 26 years in this cohort was previously published [20].

### Participants

The VLBW group was defined by birthweight ≤1500 g and all were born preterm. Of the 121 VLBW children, 33 infants died in the neonatal period. Exclusion criteria at birth were syndromes or congenital malformations (two excluded). Further two with severe quadriplegia and mental retardation were excluded at follow-up (not testable). Hence, 84 individuals were eligible for the study and invited, of whom 61 (73%) participated (33 males, 28 females).

The SGA group was born at term with birthweight <10th percentile adjusted for gestational age, sex and parity. Among the 1249 eligible women, 104 (8%) gave birth to a SGA child at term. One was excluded at birth (according to criteria), one died before follow-up and one was excluded at follow-up (not testable). Of the 101 eligible, one could not be reached, and 68 (67% of eligible) participated (33 males, 35 females).

The control group was born at term with birthweight ≥10th percentile. This group comprised 120 children, born to mothers in the 10% random sample. Two were excluded according to exclusion criteria at birth. Of the 118 eligible, two could not be reached, and 88 (75% of eligible) participated (38 males, 50 females).

### Non-participants

Non-participant characteristics are presented in Additional file 1: Table S1. Compared to participants, VLBW non-participants had lower birthweight and SGA non-participants had lower IQ score at 19 years and lower parental socioeconomic status (SES).

### Clinical measures

The Achenbach System of Empirically Based Assessment, Adult Self-Report [21] (ASEBA ASR) was used to assess adaptive functioning and problems during the last 6 months. ASEBA ASR is a comprehensive, self-administered questionnaire with 126 problem items (rated 0; “not true”, 1; “sometimes true”, 2; “often true”) generating eight syndrome scales: Anxious/Depressed, Withdrawn, Somatic Complaints, Thought Problems, Attention Problems, Aggressive Behavior, Rule-Breaking Behavior and Intrusive. Three composite scales are computed: Total Problems (all problem items), Internalizing (Anxious/Depressed, Withdrawn and Somatic Complaints) and Externalizing Problems (Aggressive Behavior, Rule-Breaking Behavior and Intrusive). A Critical Items scale denotes items indicating severe psychiatric symptoms of particular clinical relevance, i.e. hallucinations, suicidal thoughts etc. Higher score indicates more problems. Borderline/clinical level was defined by raw score ≥ 93rd percentile in the control group for problem scores and ≥ 84th percentile for composite scales, according to the manual.

The Autism-Spectrum Quotient (AQ) [22] is a self-administered questionnaire designed for adults with normal intelligence (IQ ≥85) and consists of 50 items assessing five domains of autism spectrum traits: Social skill, Attention switching, Attention to detail, Communication and Imagination. It is scored as 0 for “do not agree” or 1 for “mildly or strongly agree”. A score of 20–31 denotes intermediate level and ≥ 32 denotes high level autism spectrum traits.

Peters et al. Delusions Inventory (PDI) [23, 24] was used for assessing odd beliefs and mild delusions. It is a self-report questionnaire with 21 items covering multiple categories of delusional ideation. PDI gives a Yes/No score, three dimension scores for Distress, Preoccupation and Conviction, with single items ranging from a low of 0 to a high of 5, and a Grand Total score summing all scores. Higher score indicates more symptoms.

Perinatal risk factors included birthweight, gestational age at birth, days admitted to the NICU or pediatric ward, days with respiratory support (ventilator or continuous positive airway pressure), presence of IVH on neonatal cerebral ultrasound, prenatal maternal glucocorticoids and Apgar score at 5 min.

To assess childhood motor function the Psychomotor Development index of the Bayley Scales of Infant Development, first edition (BSID) [25] with age-adjusted standard scores from 1 year of age was used (corrected age for the VLBW group). At 5 years, three sub-scores of the Peabody Developmental Motor Scales (PDMS) [26] were used: Eye-hand coordination, Balance and Locomotor. Lower scores on the BSID and PDMS indicate more motor problems. At 14 years, the Movement Assessment Battery for Children (Movement ABC) [27] total score was used. Higher score on Movement ABC indicates more motor problems.

The Wechsler Preschool and Primary Scale of Intelligence (WPPSI) [28] was used to calculate full IQ at 5 years, the Wechsler Intelligence Scale for Children – 3rd edition (WISC-III) [29] four subsets (Vocabulary, Arithmetic, Block design and Picture arrangement) was used at 14 years and the full Wechsler Adult Intelligence Scale 3rd edition (WAIS-III) [30] was applied at 19 years.

Parental SES (ranging from low of 1 to high of 5) was calculated according to Hollingshead’s two factor index of social position, based on information on parents’ education and occupation (adapted to today’s categories). Parental SES was collected at 14 years and supplemented at 19 years.

### Statistics

Power was estimated prior to conducting the study and was minimum 93% for the preterm VLBW group and 78% for the term SGA group. Pearson’s chi-squared test was used for comparing dichotomous outcomes between groups. Skewed ordinal and continuous outcomes were compared using nonparametric tests. In order to preserve the familywise error rate due to comparisons between three groups, we reported the maximum *p*-values of the three-group comparison and pairwise comparisons [31]. In order to assess the effect of possible confounders, we used linear regression with ASEBA ASR Total Problems raw score as the dependent variable and birthweight group as categorical covariate, and adjusted separately for each of the potential confounders: sex, parental SES, maternal age at birth, and maternal smoking at conception. We used an identical approach to confounder analysis with AQ Sum score and PDI Grand Total score as dependent variables. Possible neonatal risk factors and childhood early markers of adult psychiatric problems were assessed separately in the VLBW and the SGA group, using linear regression with the ASEBA ASR Total Problems raw score, AQ Sum score and PDI Grand Total score as dependent variables, and possible predictors as covariates, one at a time. Normality of residuals was checked by visual inspection of QQ plots. Ninety-five percent confidence intervals (CI) are reported where relevant. Two-sided *p*-values < 0.05 were considered significant. Due to multiple hypotheses, adjustments according to Benjamini and Hochberg [32] was applied for Tables 2, 3, 4, Additional file 1: Table S2 and S5. Adjustments were performed separately for each table and separately for each questionnaire within one table.

## Results

### Clinical characteristics and previous follow-up results

Clinical characteristics are presented in Table 1. Birthweight, gestational age and head circumference at birth differed between groups by study design. Mean Apgar score at 5 min was lower in the VLBW group than in the control group. IVH was visible on neonatal ultrasonography in nine (15%) VLBW participants; four (7%) grade 1, two (3%) grade 2, one (2%) grade 3 and two (3%) grade 4. Mean maternal age at birth was lower in the VLBW group (28.4 years, SD = 5.1) and the SGA group (28.6 years, SD = 3.9) compared to the control group (30.6 years, SD = 4.4), and a higher percentage of SGA mothers (59%) smoked at time of conception compared to controls (40%). CP had been diagnosed in three VLBW participants at previous follow-up. Motor scores at 1 and 5 years did not differ significantly between the VLBW or the SGA group and the control group. Mean Movement ABC total score at 14 years was higher in the VLBW group compared to the control group. When we excluded VLBW participants with CP, the mean (SD) total score of Movement ABC was reduced from 11.6 (8.4) to 10.8 (7.6) but the difference between the VLBW and control group was still statistically significant (*p* = .001). Mean IQ score was lower in both the VLBW and the SGA group compared to controls at 5 and 19 years, while at 14 years in the VLBW group only. None of the participants were blind or deaf. Mean parental SES was similar in the three study groups.

**Table 1:** Clinical characteristics of the participants in the three study groups

	Preterm VLBW (*N* = 61)	*p*	Term SGA (*N* = 68)	*p*	Control (*N* = 88)
Maternal smoking at conception, n (%)	–	–	34/58 (59)	.029	34/85 (40)
Maternal age at birth, years (*n* = 60/58/85) mean (SD)	28.4 (5.1)	.009	28.6 (3.9)	.007	30.6 (4.4)
Maternal glucocorticoid before birth, n (%)	29/59 (49)	–	–	–	–
Birth characteristics, mean (SD)					
Birthweight, g	1203 (247)	–	2938 (193)	–	3702 (451)
Gestational age, weeks	28.9 (2.7)	–	39.6 (1.2)	–	39.8 (1.2)
Head circumference, cm (*n* = 49/57/85)	27.0 (2.5)	–	33.8 (1.1)	–	35.4 (1.1)
Male sex, n (%)	33/61 (54)	.420	33/68 (49)	.420	38/88 (43)
Twins, n (%) ^a^	9/61 (15)	–	0 (0)	–	0 (0)
Perinatal factors, mean (SD)					
Apgar at 5 min (*n* = 59/57/83)	8.3 (1.8)	<.001	9.9 (0.3)	.564	9.8 (1.0)
Days in NICU (*n* = 58/−/−) ^b^	67.6 (32.0)	–	–	–	–
Days with respiratory support (*n* = 60/−/−) ^c^	5.7 (12.1)	–	–	–	–
IVH, n (%) ^d^	9/59 (15)	–	–	–	–
Motor function, mean (SD)					
BSID Psychomotor Developmental Index at 1 year (*n* = 22/48/75)	99.1 (18.4)	.141	104.4 (13.5)	.141	107.8 (11.8)
PDMS Eye/hand coordination at 5 years (*n* = 23/56/71)	79.4 (5.8)	.734	78.5 (5.1)	.033	80.9 (3.3)
PDMS Balance at 5 years (*n* = 23/57/71)	57.3 (4.5)	.286	58.3 (4.8)	.286	59.0 (4.4)
PDMS Locomotion at 5 years (*n* = 23/56/71)	100.7 (10.9)	.264	104.1 (8.6)	.264	105.9 (5.4)
Movement ABC total score, at 14 years (*n* = 41/48/68)	11.6 (8.4)	<.001	6.8 (5.3)	.770	6.0 (4.1)
CP, n (%)	3/61 (5)	–	0 (0)	–	0 (0)
Cognitive function, mean IQ (SD)					
WPSSI 5 years (*n* = 21/52/75)	94.9 (16.6)	<.001	100 (12.7)	.001	108.0 (12.7)
WISC 14 years (*n* = 44/49/69) ^e^	81.0 (21.6)	<.001	92.0 (15.8)	.171	96.2 (16.7)
WAIS 19 years (*n* = 46/46/68)	88.4 (11.9)	<.001	95.7 (9.2)	.004	101.7 (12.3)
IQ < 85, WAIS 19 years, n (%)	16/46 (35)	<.001	6/46 (13)	.347	5/68 (7)
Parental SES, mean (SD) (*n* = 50/55/74) ^f^	3.5 (1.2)	.372	3.7 (1.2)	.372	3.8 (1.1)
Age at participation, years, mean (SD)	26.4 (0.7)	.084	26.5 (0.6)	.952	26.5 (0.5)

*P*-values vs. controls

*Abbreviations*: *VLBW* Very Low Birth Weight, *SGA* Small for Gestational Age, *NICU* Newborn Intensive Care Unit, *IVH* intraventricular hemorrhage, *BSID* Bayley Scales of Infant Development, *PDMS* Peabody Developmental Motor Scales, *CP* Cerebral Palsy, *WPPSI* Wechsler Preschool and Primary Scale of Intelligence, *WISC* Wechsler intelligence scale for children, *WAIS* Wechsler Adult Intelligence Scale, *SES* Socioeconomic Status

^a^ From 8 pairs

^b^ Information on days in NICU or pediatric ward: *n* = 58 VLBW, max.: 160 days

^c^ Information on days with respiratory support (Ventilator or Continuous Positive Airway Pressure): *n* = 60 (No respiratory support: *n* = 22 (37%); One day respiratory support *n* = 15 (25%) max: 63 days; among those with respiratory support ≥1 day: *n* = 23 (38%), mean (SD): 9,0 days (14.2))

^d^ IVH Grading: No IVH: 50 (85%), grade 1: *n* = 4; grade 2: *n* = 2, grade 3: *n* = 1; grade 4: *n* = 2

^e^ WISC estimated from 4 subtests

^f^ Parental SES collected at 14 years for: *n* = 47 VLBW, 53 SGA, 72 control and supplemented at 19 years for *n* = 3 VLBW, 2 SGA, 2 control

### Psychiatric symptom load

**Fig. 2:**
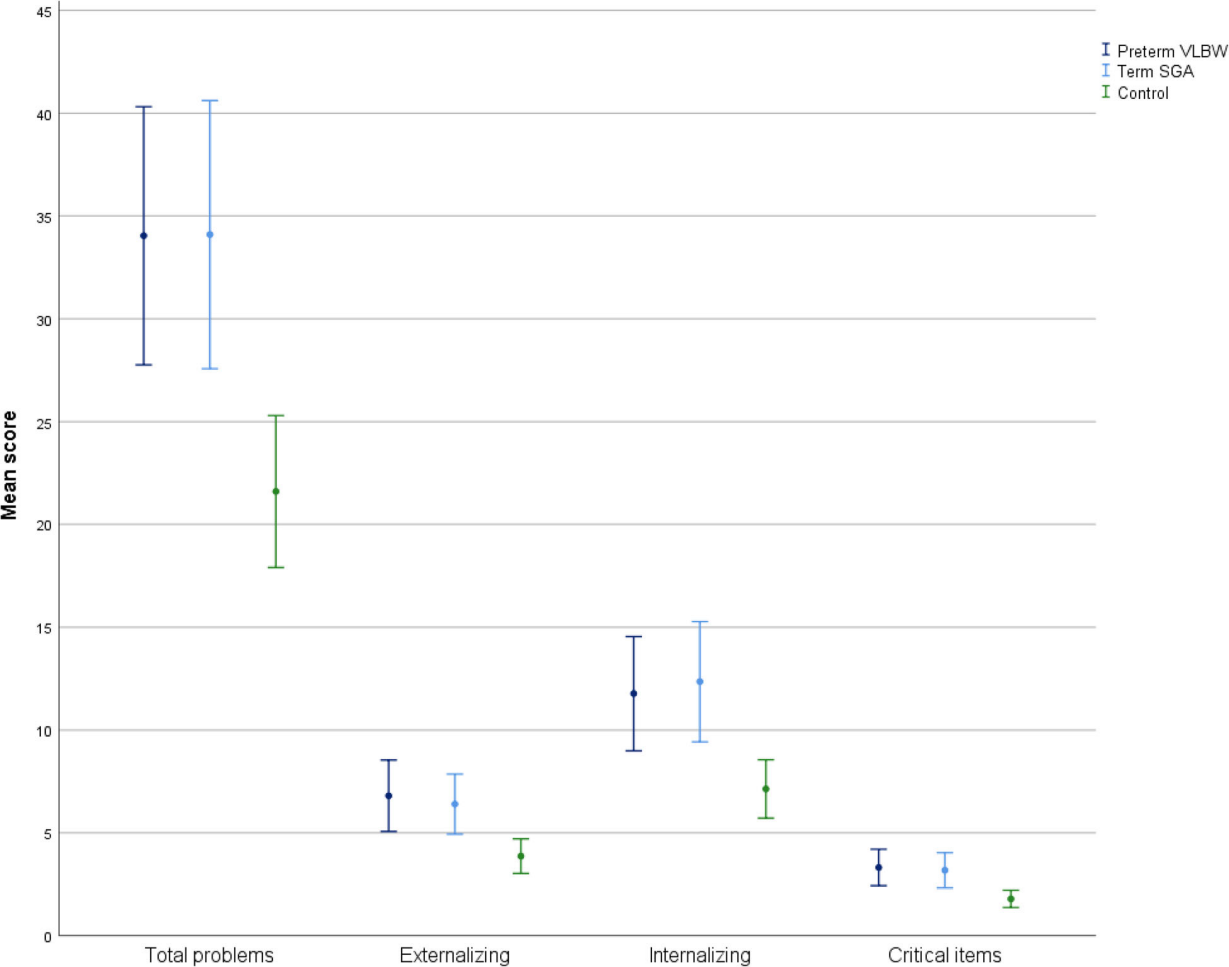
Error bars ASEBA ASR Composite scores. Error bars are 95% Confidence Intervals

**Fig. 3:**
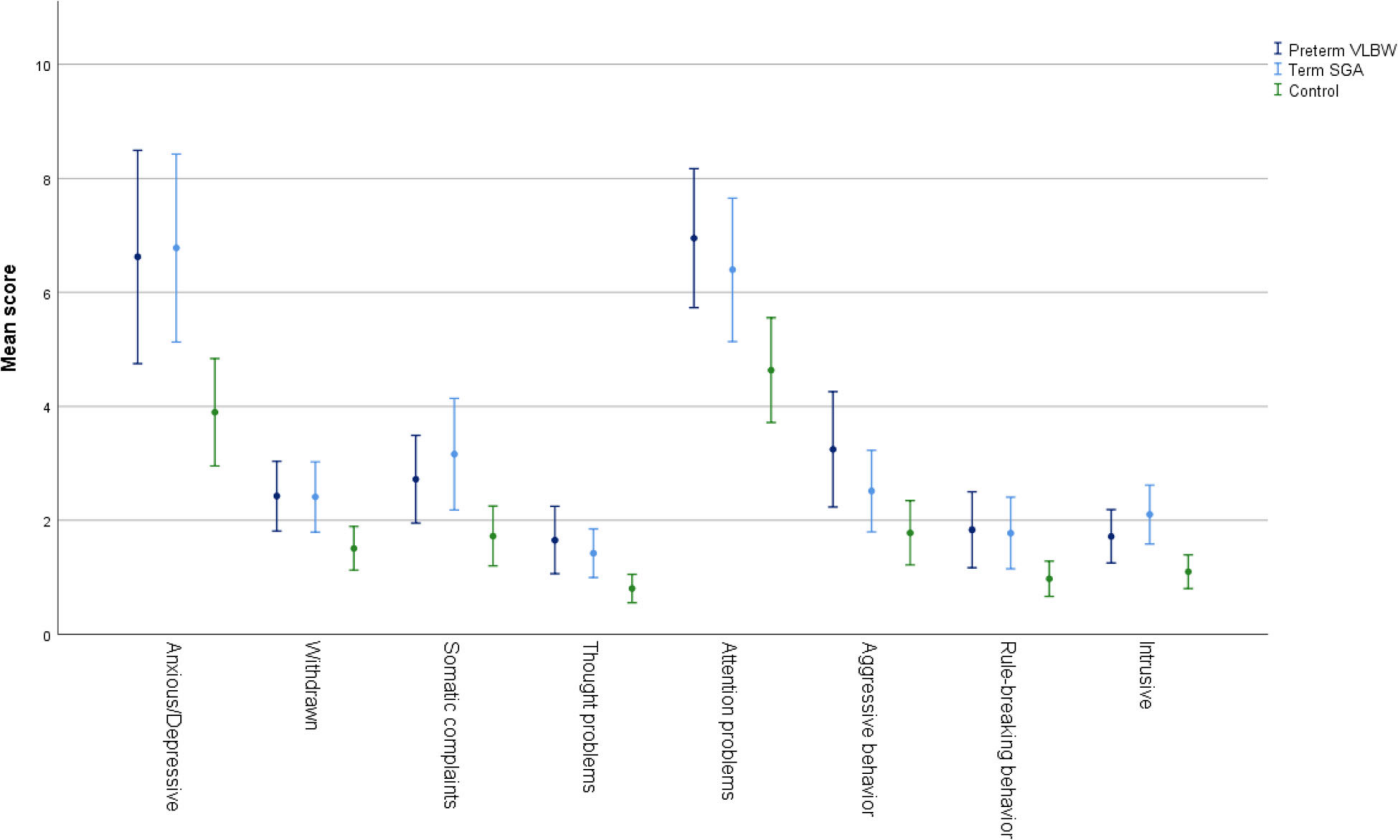
Error bars ASEBA ASR Problem scores. Error bars are 95% Confidence Intervals

*The VLBW group* had higher mean score on ASEBA ASR Total Problems compared to the control group; mean (SD): 34.0 (24.5) vs 21.6 (17.4), Cohens d = 0.71, *p* = .012 (Table 2, Fig. 2). They also had higher mean scores on Externalizing Problems as well as Withdrawn, Somatic Complaints, Thought Problems, Attention Problems, Intrusive and Critical Items, with small to medium effect sizes (Cohens d) (Figs. 2 and 3). A higher proportion of VLBW participants were in borderline/clinical range for Total Problems as well as Internalizing and Attention Problems compared to controls (Table 3).

**Table 2:** Psychiatric symptom load in the three study groups

	Preterm VLBW (*N* = 61)		*p*	Term SGA (*N* = 68)		*p*	Control (*N* = 88)
	Mean (SD)	*Cohen’s d*		Mean (SD)	*Cohen’s d*		Mean (SD)
ASEBA Raw Scores							
Anxious/Depressed	6.6 (7.3)	0.60	.069	6.8 (6.8)	0.64	.039	3.9 (4.5)
Withdrawn	2.4 (2.4)	0.50	.032	2.4 (2.5)	0.50	.047	1.5 (1.8)
Somatic Complaints	2.7 (3.0)	0.40	.032	3.2 (4.1)	0.60	.074	1.7 (2.5)
Thought Problems	1.7 (2.3)	0.75	.032	1.4 (1.8)	0.50	.039	0.8 (1.2)
Attention Problems	7.0 (4.8)	0.56	.015	6.4 (5.2)	0.42	.042	4.6 (4.3)
Aggressive Behavior	3.3 (4.0)	0.56	.062	2.5 (3.0)	0.26	.078	1.8 (2.7)
Rule-Breaking Behavior	1.8 (2.6)	0.53	.062	1.8 (2.6)	0.53	.078	1.0 (1.5)
Intrusive	1.7 (1.8)	0.43	.037	2.1 (2.1)	0.71	.016	1.1 (1.4)
Internalizing Problems	11.8 (10.8)	0.70	.032	12.4 (12.1)	0.79	.039	7.1 (6.7)
Externalizing Problems	6.8 (6.6)	0.73	.015	6.4 (6.0)	0.63	.016	3.9 (4.0)
Total Problems	34.0 (24.5)	0.71	.012	34.1 (27.0)	0.72	.016	21.6 (17.4)
Critical Items	3.3 (3.5)	0.75	.015	3.2 (3.5)	0.70	.039	1.8 (2.0)
AQ ^a^							
Social skill	2.0 (1.7)	0.47	.028	2.1 (2.3)	0.53	.136	1.3 (1.5)
Attention switching	4.4 (2.2)	0.55	.012	4.3 (2.1)	0.50	.012	3.3 (2.0)
Attention to detail	3.5 (2.1)	0.18	.395	3.4 (1.9)	0.14	.395	3.1 (2.2)
Communication	2.4 (1.7)	0.35	.156	2.3 (2.0)	0.29	.156	1.8 (1.7)
Imagination	3.2 (1.8)	0.29	.496	2.8 (1.6)	0.00	.496	2.8 (1.4)
Sum score	15.5 (6.3)	0.60	.028	14.6 (6.1)	0.43	.136	12.3 (5.3)
PDI ^b^							
Conviction	5.9 (7.9)	0.23	.590	5.1 (5.8)	0.11	.590	4.4 (6.6)
Preoccupation	3.8 (5.6)	0.29	.454	3.7 (5.7)	0.26	.454	2.6 (4.2)
Distress	4.2 (5.9)	0.27	.454	3.9 (5.9)	0.20	.454	3.0 (4.5)
Grand Total	15.7 (21.2)	0.27	.454	14.3 (17.7)	0.18	.454	11.3 (16.5)
Yes/no	1.9 (2.3)	0.29	.454	1.7 (1.7)	0.18	.454	1.4 (1.7)

*P* values vs control by non-parametric test, adjusted for multiple comparison according to Benjamini and Hochberg [32] and for three group comparison according to Levin et al. [31]

*Abbreviations*: *VLBW* Very Low Birth Weight, *SGA* Small for Gestational Weight, *AQ* Autism-Spectrum Quotient, *PDI* Peters et al. Delusions Inventory

^a^ AQ Sum score: *n* = 59 VLBW, 65 SGA, 85 control; Social Skill Subscale and Attention switching: *n* = 59 VLBW, 66 SGA, 87 Control; Attention to detail: *n* = 60 VLBW, 67 SGA, 87 Control; Communication: *n* = 59 VLBW, 66 SGA, 85 Control; Imagination: *n* = 59 VLBW, 65 SGA, 87 Control

^b^ PDI: *n* = 56 VLBW, 66 SGA, 86 control

**Table 3:** Number and percent of participants with borderline/clinical range symptom level in the three study groups

	Preterm VLBW	*p*	Term SGA	*p*	Control
	n/N (%)		n/N (%)		n/N (%)
ASEBA ASR borderline/clinical range					
Anxious/Depressed	12 /61 (20)	.070	15 /68 (22)	.100	7 /88 (8)
Withdrawn	13 /61 (21)	.182	18 /68 (27)	.115	11 /88 (13)
Somatic Complaints	9 /61 (15)	.201	15 /68 (22)	.100	7 /88 (8)
Thought Problems	13 /61 (21)	.219	13 /68 (19)	.256	10 /88 (11)
Attention Problems	15 /61 (25)	.042	14 /68 (21)	.100	7 /88 (8)
Aggressive Behavior	13 /61 (21)	.070	8 /68 (12)	.284	6 /88 (7)
Rule-Breaking Behavior	14 /61 (23)	.182	13 /68 (19)	.224	10 /88 (11)
Intrusive	10 /61 (16)	.155	15 /68 (22)	.100	7 /88 (8)
Internalizing Problems	26 /61 (43)	.007	24 /68 (35)	.063	14 /88 (16)
Externalizing Problems	21 /61 (34)	.096	23 /68 (34)	.100	17 /88 (19)
Total Problems	23 /61 (38)	.023	23 /68 (34)	.063	14 /88 (16)
Critical Items	14 /61 (23)	.096	17 /68 (25)	.100	10 /88 (11)
AQ ≥ 20 point	21 /59 (36)	.007	13 /65 (20)	.100	8 /85 (9)
PDI ≥ 4 yes	13 /56 (23)	.025	7 /66 (11)	.284	5/ 86 (6)

*P* values vs control by chi square test, adjusted for multiple comparison according to Benjamini and Hochberg [32] and for three group comparison according to Levin et al. [31]

*Abbreviations*: *VLBW* Very Low Birth Weight, *SGA* Small for Gestational Age, *ASEBA ASR* The Achenbach System of Empirically Based Assessment, Adult Self Report, *AQ* Autism-Spectrum Quotient, *PDI* Peters et al. Delusions Inventory

ASEBA ASR borderline/ clinical level defines as >84th percentile (total problems, internalizing, externalizing) and > 93rd percentile (problem scores) in the control group

The AQ Attention switching score was higher in the VLBW group compared to controls; 4.4 (2.2) vs 3.3 (2.0), *p* = .012, as well as higher scores in the mean Sum score and the subscale Social skill (Table 2). Effect sizes were small to medium. When we excluded VLBW participants with IQ below 85, these results were no longer statistically significant (Additional file 1: Table S2). A higher number of VLBW participants had an AQ Sum score ≥ 20 points than in the control group: 21 of 59 (36%) vs 8 of 85 (9%), *p* = .007 (Table 3). When we excluded VLBW participants IQ < 85, 8 of 30 (27%) VLBW, vs 2 of 60 (3%) controls, *p* = .035, had a Sum score ≥ 20 points (Additional file 1: Table S2). None had Sum score ≥ 32 points.

The higher PDI Grand Total score in the VLBW group compared with the control group was not statistically significant; 15.7 (21.2) vs 11.3 (16.5), *p* = 0.454, nor were the subscales Conviction, Preoccupation nor Distress scores (Table 2). Effect sizes were small. Still, a higher number of VLBW participants had Yes-score ≥ 4 than controls; 13 of 56 (23%) vs 5 of 86 (6%) *p* = .025 (Table 3).

When we adjusted for possible confounders, the regression coefficients for VLBW vs controls remained approximately unchanged for ASEBA ASR Total Problems score, AQ Sum score and PDI Grand Total score (Additional file 1: Table S3).

*The SGA group* had significantly higher mean score on ASEBA ASR Total Problems compared to the control group: 34.1 (27.0) vs 21.6 (17.4), Cohens d = 0.72, *p* = .016 (Table 2, Fig. 2). They also had higher scores on Externalizing and Internalizing Problems as well as Anxious/Depressed, Withdrawn, Thought Problems, Attention Problems, Intrusive and Critical Items, with medium effect sizes (Figs. 2 and 3). The number of SGA participants in borderline/clinical range was non-significantly higher compared with controls for Total Problems and Internalizing Problems (Table 3).

The AQ Attention switching score was higher in the SGA group compared to the control group: 4.3 (2.1) vs 3.3 (2.0), *p* = .012 (Table 2). Effect sizes were small to medium. After excluding participants with IQ < 85, the group difference in Attention switching remained statistically significant (Additional file 1: Table S2). The frequency of SGA participants with Sum score ≥ 20 points was 13 of 65 (20%) vs eight of 85 (9%) control participants, *p* = .100 (Table 3), and after exclusion of participants with IQ < 85, 5 of 38 (13%) vs 2 of 60 (3%), *p* = .368 (Additional file 1: Table S2). None had Sum score ≥ 32 points.

On the PDI, differences between the SGA group and the control group were not statistically significant (Table 3).

When we adjusted for possible confounders, the regression coefficients for SGA vs controls remained approximately unchanged for ASEBA ASR Total Problems, AQ Sum score and PDI Grand Total score (Additional file 1: Table S4).

### Perinatal risk factors and early neurodevelopmental markers

In the preterm born VLBW group, increasing number of days spent in NICU was associated with more overall psychiatric symptoms (ASEBA ASR Total Problems score), autistic traits (AQ Sum score) and odd beliefs (PDI Grand Total score) (Table 4). Lower gestational age and increasing number of days with respiratory support were associated with more overall psychiatric symptoms and autistic traits, while presence of IVH was associated with more autistic traits. The corresponding adjusted R^2^ values were of small to moderate size (0.078 to 0.169). Poor motor function at 14 years was associated with more overall psychiatric symptoms, autistic traits and odd beliefs (adjusted R^2^ 0.078 to 0.175). The associations surviving adjustment for multiple comparisons were those between increasing number of days spent in NICU as well as increasing number of days with respiratory support and more overall psychiatric problems as well as autistic traits. Also the association between motor problems at 14 years and more overall psychiatric problems survived adjustment for multiple comparison. When we excluded VLBW participants with CP (Additional file 1: Table S5), the regression coefficients for overall psychiatric symptoms and autistic traits as dependent variables were essentially unchanged when adjusting for days spent in NICU and days with respiratory support, but was reduced when adjusting for gestational age and motor function. The regression coefficients for odd beliefs were essentially unchanged when adjusting for days spent in NICU, reduced for motor function, but statistically significant for days with respiratory support and presence of IVH. After adjustment for multiple comparison, none of these coefficients was statistically significant.

**Table 4:** Perinatal risk factors and neurodevelopmental markers in the preterm VLBW and the term SGA group; linear regression with ASEBA ASR Total Problems score, AQ Sum score and PDI Grand Total score separately as dependent variables, and for each variable one at a time as covariate

	ASEBA ASR	*p*	AQ	*p*	PDI	*p*
	B (95% CI)		B (95% CI)		B (95% CI)	
Preterm VLBW						
Perinatal factors						
Gestational Age (weeks, *n* = 61/59/60)	−2.4 (−4.7 to − 0.05)	.161	− 0.7 (−1.3 to − 0.1)	.102	− 1.7 (−3.7 to 0.4)	.259
Birthweight (pr. 100 g, *n* = 61/59/60)	− 2.2 (− 4.7 to 0.4)	.184	− 0.5 (− 1.2 to 0.1)	.212	− 2.0 (− 4.2 to 0.2)	.259
Days in NICU (*n* = 58/56/58)	0.3 (0.1 to 0.5)	.014	0.08 (0.03 to 0.12)	.028	0.2 (0.04 to 0.4)	.224
Days with respiratory support (*n* = 60/58/59) ^a^	0.9 (0.4 to 1.4)	.014	0.2 (0.06 to 0.3)	.028	0.4 (−0.1 to 0.8)	.259
IVH (*n* = 59/57/58)	4.1 (−3.1 to 11.4)	.330	1.8 (0.03 to 3.6)	.129	4.8 (−1.5 to 11.1)	.266
Maternal glucocorticoid (*n* = 59/57/58)	−4.4 (−17.2 to 8.4)	.525	−0.8 (−4.1 to 2.5)	.736	−5.6 (− 17.0 to 5.9)	.466
Apgar 5 min (*n* = 59/57/58)	−2.3 (− 5.9 to 1.4)	.308	−0.6 (− 1.5 to 0.4)	.425	− 1.5 (− 4.7 to 1.8)	.467
Motor function						
BSID Psychomotor Developmental Index score at 1 year (*n* = 22/21/22)	− 0.2 (− 0.7 to 0.4)	.525	− 0.1 (− 0.2 to 0.1)	.575	0.1 (− 0.5 to 0.6)	.813
PDMS Eye/hand coordination at 5 years (*n* = 23/22/23)	− 1.7 (− 3.5 to 0.2)	.184	−0.1 (− 0.6 to 0.4)	.736	− 1.7 (− 3.7 to 0.2)	.259
PDMS Balance at 5 years (*n* = 23/22/23)	−2.1 (− 4.4 to 0.3)	.184	−0.2 (− 0.9 to 0.4)	.575	−1.8 (− 4.4 to 0.8)	.305
PDMS Locomotion at 5 years (*n* = 23/22/23)	−0.7 (− 1.7 to 0.3)	.258	0.1 (− 0.4 to 0.1)	.473	−0.6 (− 1.7 to 0.5)	.446
Movement ABC total score at 14 years (*n* = 41/39/40)	1.2 (0.4 to 2.0)	.019	0.3 (0.05 to 0.5)	.089	0.8 (0.02 to 1.7)	.259
Cognitive function						
WPSSI at 5 years (*n* = 21/20/21)	−0.2 (−0.8 to 0.4)	.525	−0.02 (− 0.2 to 0.2)	.803	0.1 (− 0.4 to 0.7)	.728
WISC at 14 years (*n* = 44/42/43) ^b^	−0.2 (− 0.5 to 0.1)	.258	−0.1 (− 0.2 to 0.2)	.212	−0.1 (− 0.4 to 0.2)	.748
Term SGA						
Perinatal factors						
Gestational Age (weeks, *n* = 68/65/68)	−6.1 (−11.5 to −0.7)	.290	−1.0 (−2.3 to 0.3)	.270	−3.1 (−7.1 to 0.8)	.285
Birthweight (pr. 100 g, *n* = 68/65/68)	−1.6 (− 5.0 to 1.8)	.778	−0.3 (− 1.1 to 0.5)	.514	− 1.2 (− 3.7 to 1.2)	.515
Apgar 5 min (*n* = 57/55/57)	16.7 (− 11.8 to 45.1)	.778	− 0.8 (− 6.9 to 5.4)	.806	4.6 (− 16.5 to 25.8)	.734
Motor function						
BSID Psychomotor Developmental Index score at 1 year (*n* = 48/47/48)	− 0.1 (− 0.7 to 0.5)	.883	− 0.1 (− 0.2 to 0.02)	.270	0.1 (− 0.4 to 0.6)	.734
PDMS Eye/hand coordination at 5 years (*n* = 56/54/56)	−0.4 (− 1.7 to 1.0)	.778	− 0.3 (− 0.7 to − 0.01)	.215	−1.0 (− 2.1 to 0.01)	.283
PDMS Balance at 5 years (*n* = 57/54/57)	−0.5 (− 1.9 to 0.9)	.778	− 0.1 (− 0.5 to 0.3)	.746	−0.6 (− 1.8 to 0.5)	.515
PDMS Locomotion at 5 years (*n* = 56/54/56)	− 0.2 (− 1.0 to 0.6)	.778	− 0.2 (− 0.3 to 0.05)	.270	−0.03 (− 0.7 to 0.6)	.929
Movement ABC total score at 14 years (*n* = 48/45/48)	0.4 (−1.2 to 1.9)	.778	0.2 (−0.2 to 0.6)	.359	0.5 (−0.7 to 1.8)	.559
Cognitive function						
WPSSI at 5 years (*n* = 52/50/52)	−0.4 (−1.0 to 0.2)	.778	−0.2 (− 0.4 to − 0.1)	.010	−0.4 (− 0.9 to 0.02)	.283
WISC at 14 years (*n* = 49/46/49) ^b^	− 0.01 (− 0.5 to 0.5)	.956	−0.1 (− 0.2 to 0.04)	.288	−0.3 (− 0.7 to 0.05)	.283

*P* values adjusted for multiple comparisons according to Benjamini and Hochberg [32]

*Abbreviations*: *VLBW* Very Low Birth Weight, *NICU* Neonatal Intensive Care Unit, *IVH* Intra Ventricular Hemorrhage, *BSID* Bayley Scales of Infant Development, *PDMS* Peabody Developmental Motor Scales, *WPPSI* Wechsler Preschool and Primary Scale of Intelligence, *WISC* Wechsler intelligence scale for children

^a^ Respiratory support: Ventilator or Continuous Positive Airway Pressure

^b^ WISC estimated from 4 subtests

In the term-born SGA group, lower gestational age was associated with more overall adult psychiatric symptoms (ASEBA ASR Total score), although the corresponding adjusted R^2^ was small (≤ 0.10) (Table 4). Poorer eye/hand coordination at 5 years and lower IQ at 5 years were associated with more autistic traits (AQ Sum score), with adjusted R^2^: 0.059 and 0.194 respectively. After adjustment for multiple comparison, the association between lower IQ at 5 years and more adult autistic traits was still statistically significant.

## Discussion

At 26 years of age, both the preterm VLBW and the term SGA group had more overall self-reported psychiatric symptoms compared to the term non-SGA control group. The VLBW and the SGA group were similar in terms of reporting more attention, internalizing and externalizing problems. However, the VLBW group reported more somatic complaints and a higher proportion had intermediate level of autistic traits, while the SGA group reported more anxious/depressed symptoms compared to controls. Effect sizes (Cohens d) were of small to medium size. In the VLBW group, increasing number of days spent in NICU and with respiratory support were perinatal risk factors, while poor motor function in early adolescence seemed to be a neurodevelopmental marker.

### Symptom load

In the preterm VLBW group, the number of participants with borderline/clinical symptom level was consistently higher across symptom categories compared to controls. Put together with the higher total problems and critical items scores, this indicates a substantial burden of mental health problems.

Our results are in line with a recent meta-analysis by Pyhälä et al. [33], reporting more internalizing problems in VLBW adults compared to their term-born peers. Internalizing problems include anxiety and depression, which are well documented in VLBW individuals both in this and other cohorts [8, 20, 34]. However, we found that anxiety and depression were less evident by self-report than by the clinical diagnostic assessment published previously [20]. Either, this discrepancy could be due to the young adults under-reporting symptoms of anxiety or that their disorders were well managed.

The externalizing problems reported in this study, is contradictory to other studies [33, 35], and surprisingly, a quite high proportion of VLBW participants reported aggressive behavior in the clinical range. The ASEBA ASR Aggression Behavior scale contains items related mostly to verbal or relational aggression, as well as items concerning mood changes and instability, and may thus be related to anxiety and depression, and possibly bipolar disorder, which was found with increased prevalence in this VLBW group [20]. Further, subjective measures on anger and aggression have been related to psychiatric diagnoses and symptoms in general [36]. One may also speculate whether the self-perceived aggression relates to the lower cognitive abilities and attention problems in the VLBW group, which may impede mental construction of a predictable and understandable reality, causing slight confusion and aggression.

Attention problems was a conspicuous feature in the VLBW group, and is well-known from the literature where it rarely is accompanied by hyperactivity or impulsivity. Attention deficit is likely to represent a challenge in academic performance and everyday life, and attention problems and perhaps especially difficulties in attention switching, may negatively influence social functioning, particularly in group settings. This is supported by the poorer social skills reported by VLBW participants and the higher frequency of intermediate level of autistic traits compared to controls. These results imply difficulties in understanding social cues and limited flexibility in social situations. Elevated rates of autistic traits in children born preterm are consistently reported [37], also when excluding participants with severe impairments [38]. Our findings indicate that the attention- and social problems of the VLBW group seem not to subside with advancing age, but continue into adulthood.

Almost one in four VLBW individuals reported multiple odd beliefs according to PDI, and even though the higher mean scores were not significantly different from controls, this finding might indicate a higher frequency of strange ideas or less ability to suppress and rationalize such ideas.

In all, when reviewing the VLBW symptom profile in this cohort, the diversity is striking, painting a broader picture of a rather complex phenotype, possibly reflecting aberrant brain development with a diffuse global influence on mental health.

The term-born SGA group reported overall higher level of psychiatric symptoms than the control group. The degree of attention and attention switching problems was interestingly similar to the preterm VLBW group. Increased risk of hyperkinetic disorder has been reported in term-born SGA children [39], hence it seems such difficulties continue into adult age, and may possibly account for the lower educational attainment seen in this group [20, 40]. Emotional problems were prevalent in the SGA group, supporting the results of Berle et al. [41] who found increased risk of anxiety and depressive symptoms in term-born SGA adults. Emotional and behavioral problems were however not seen by Cornforth et al. [42] in preschool children, possibly indicating that other aspects, such as genetics or environmental factors, are influential for adult psychiatric symptoms in this group, or perhaps an age specific expression of problems. Almost one in four had borderline/clinical level of Somatic Complaints in the SGA group, and a higher level of self-reported pain compared to normal birthweight peers has been reported [43]. Both pain and other somatic complaints may be related to emotional difficulties such as anxiety and depression, but could also be connected to the well-established relationship between early life exposures and adult somatic disorders in general [44]. Intrusive problems were a prominent feature in the SGA group, indicating less self-regulation of behavior and emotions, and poorer social skills. Further, although not statistically significant, the higher proportion with intermediate level of autistic traits in the term SGA group call for more research.

Despite the overall more moderate severity of the symptom profile in the SGA group, the similarities to the preterm VLBW group is noteworthy. Being born SGA is closely related to fetal growth restriction, but will also include genetically small individuals, leaving the reason for smallness even more important. We speculate that the similarities may be related to some shared prenatal exposure or developmental course.

### Perinatal risk and neurodevelopmental markers

In the VLBW group, lower gestational age and increasing number of days with respiratory support and days of stay in the NICU were risk factors for adult psychiatric symptoms (although lower gestation age was non-significant after adjustment for multiple comparison). These factors are closely related to both immaturity and overall neonatal morbidity. Several studies from the same cohort have shown associations between gestational age, neonatal morbidity and brain pathology in grey and white matter [45–47], as well as associations between cerebral structural differences and psychiatric symptoms in adolescence [48]. From a clinical viewpoint, it is not surprising that the youngest and sickest babies had an increased risk of adult mental health problems. The lack of clear association between birthweight and adult psychiatric problems might be related to the inclusion criteria used in this study, with a cut-off for birthweight (≤1500 g). As the cohort was born in 1986–88 fewer babies with birthweight < 1000 g survived, resulting in a skewed distribution and thereby possibly masking an association.

There was no protective effect of prenatal steroids in this study, despite that it has been shown to be rather successful in prevention of neonatal respiratory problems [49] and one could thus expect a beneficial effect also on long-term outcome. Van Lieshout et al. [50] report increased risk of psychiatric disorders in adults exposed to prenatal corticosteroids. Postnatal steroids, given to alleviate ventilator weaning and prevent bronchopulmonary dysplasia, have turned out to have possible adverse neurodevelopmental side effects [51]. As cortisone is a stress-hormone and all steroids mimic this effect to some degree, there might be a need for further research on the long-term effect of pre- and postnatal steroids on mental health.

Motor problems at 14 years were related to psychiatric symptoms in VLBW adults, while motor function at 1 and 5 years were not associated. This can be explained by preschool motor tests inevitably being less specific than tests in adolescence. Further, as adolescence and puberty comes with extensive cerebral maturation, motor difficulties lingering beyond puberty [52] could actually be more predictive of adult psychopathology. Poole et al. [53] report an association between childhood motor problems and adult anxiety, depression and attention deficit, although weaker in extremely low birthweight individuals.

Contrary to our hypothesis, we did not find an association between childhood cognitive function and adult psychiatric symptoms. In line with our results, Westrupp et al. [16] found that IQ estimated at 5 years of age in a VLBW population could not predict adult psychiatric disorders. Our findings might be related to the inherent uncertainty of any cognitive test at this young age. It may also be related to the use of an abbreviated version of WISC at 14 years, leading to more uncertainty in the estimation of IQ.

In the term SGA group, lower gestational age was a predictive factor for overall psychiatric symptoms in adulthood, although not statistically significant after adjustment for multiple comparisons. As all participants in the SGA group were born at term (week 37 to 42), one could expect a beneficial effect of being born at early term for those subject to intrauterine growth restriction. However, some studies report negative effects on long-term outcome of even late prematurity (week 32 to 36, [54]), hence our result might indicate an extension of this gradient. Lower childhood cognitive function was associated with more autistic traits as adults. However, as the AQ subscale Attention switching is included in the concept of executive functions, poor childhood cognitive may indicate later problems in executive functions, rather than autistic traits per se. This may also apply to motor function, but the association between poor childhood fine motor function and more autistic traits was non-significant after adjustment. Nonetheless, these results call for more research, as they may imply a possibility of early detection of individuals in need of tailored follow-up in school and social settings.

### Limitations

The sample size was limited, which may influence the external validity of our findings. Thus, random findings may occur and negative findings should be interpreted with caution. Especially, the limited sample size restricted sub-analyses of SGA vs. Appropriate for Gestational Age status in the VLBW group, as well as exploring sex differences. Further, there was a lower number of participants at 1- and 5-year follow-up in the VLBW group since only those born in 1988 were enrolled, causing reduced power in the analysis of childhood cognitive and motor function. In addition, the motor tests hold different characteristics: BSID Psychomotor Developmental Index and PDMS assess the optimal motor performance, while Movement ABC seeks to identify problem areas. Self-reports were used to estimate psychiatric symptoms, and as cognitive abilities are pertinent in self-administered questionnaires, the lower IQ in the VLBW and SGA group may influence results. There were differences between participants and non-participants in birthweight (VLBW group), maternal age at birth and parental SES (SGA group), however, in the confounder analysis neither of the variables influenced the results. Apart from parental SES, we do not have information on childhood environmental factors, nor from parents or partners, which would have complemented the picture. The medical treatment and nursing of newborns has improved substantially over the nearly 30 years since this study commenced, as has public health measures, such as prevalence of smoking. This may affect the applicability of our results for more recently born VLBW infants, but results may still be relevant to infants born at the present border of viability.

This study underlines that knowledge about adult difficulties is important in order to plan for and enable tailored follow-up and also for possible early intervention. Further, low birthweight in the context of this study can be viewed as a proxy for unfavorable exposures in fetal life [55]. However, the term-born SGA group also comprised constitutionally small but otherwise healthy individuals, leaving the reasons for smallness more important for neurodevelopmental outcome. This study therefore reinforces that continued efforts for improving maternal health is warranted. For the VLBW group, the additional burden of a complicated perinatal period should further raise the awareness of possible later difficulties. Additionally, there is reason to believe that identifying motor problems in VPT/VLBW children, could be helpful in focusing extended health-care services to those with the highest risk of later unfavorable outcome.

## Conclusions

Both the preterm VLBW and the term SGA adults reported more attention, internalizing and externalizing problems and symptoms indicative of poorer social skills, compared to the controls. Increased neonatal morbidity and possibly lower gestational age were perinatal risk factors, whereas motor problems in early adolescence seemed to be a neurodevelopmental marker of adult psychiatric symptoms in the VLBW group. Our findings lend support to the idea that low birthweight involve vulnerability to neurodevelopmental disadvantages involving motor, cognitive and mental health problems. Further, symptoms seem to be expressed according to advancing age and persist into adult life.

## Additional file

Additional file 1: **Table S1.** Non-participant characteristics. **Table S2.** Descriptive values of Autism-Spectrum Quotient excluding participants with IQ < 85 at 19 years. **Table S3.** *Confounder analysis*: Linear regression coefficients with ASEBA ASR Total Problems score, AQ sum score and PDI Grand Total Score as dependent variables in the **preterm VLBW** group vs the control group, adjusted separately for each variable. **Table S4.** *Confounder analysis*: Linear regression coefficients with ASEBA ASR Total Problems score, AQ sum score and PDI Grand Total Score as dependent variables in the **term SGA** group vs the control group, adjusted separately for each variable. **Table S5.** Perinatal risk factors and neurodevelopmental markers in the preterm VLBW group excluding three participants with CP; linear regression with ASEBA ASR Total Problems score, AQ Sum score and PDI Grand Total score separately as dependent variables, and for each variable one at a time as covariate. (DOCX 41 kb)

## Data Availability

All data generated or analyzed in this study is included in this manuscript and its supplementary files. The raw data are not publicly available due to them containing information that could compromise research participants’ privacy or consent, but may in part be available from the corresponding author on reasonable request.

## Abbreviations

AQ: Autism-Spectrum Quotient
ASD: Autism spectrum disorder
ASEBA ASR: Achenbach System of Empirically Based Assessment, Adult Self-Report
BSID: Bayley Scales of Infant Development
NICU: Neonatal Intensive Care Unit
PDI: Peters et al. Delusion Inventory
PDMS: Peabody Developmental Motor Scales
SGA: Small for gestational age;
VLBW: Very low birthweight
WISC: Wechsler intelligence scale for children
WPPSI: Wechsler Preschool and Primary Scale of Intelligence
