# Supplementary material for: Psychiatric symptoms and risk factors in adults born preterm with very low birthweight or born small for gestational age at term
Source: BMC Psychiatry. 2019 Jul 17;19:223. doi: 10.1186/s12888-019-2202-8 (PMC6636134; doi:10.1186/s12888-019-2202-8)
Supplement: Supplementary file 1 — Table S1. Non-participant characteristics. Table S2. Descriptive values of Autism-Spectrum Quotient excluding participants with IQ < 85 at 19 years. Table S3. Confounder analysis: Linear regression coefficients with ASEBA ASR Total Problems score, AQ sum score and PDI Grand Total Score as dependent variables in the preterm VLBW group vs the control group, adjusted separately for each variable. Table S4. Confounder analysis: Linear regression coefficients with ASEBA ASR Total Problems score, AQ sum score and PDI Grand Total Score as dependent variables in the term SGA group vs the control group, adjusted separately for each variable. Table S5. Perinatal risk factors and neurodevelopmental markers in the preterm VLBW group excluding three participants with CP; linear regression with ASEBA ASR Total Problems score, AQ Sum score and PDI Grand Total score separately as dependent variables, and for each variable one at a time as covariate. (DOCX 41 kb) [file 12888_2019_2202_MOESM1_ESM.docx]

Additional file 1

**Table S1** Non-participant characteristics

|  | **Preterm VLBW** (N=23) | p | **Term SGA** (N=32) | p | **Control** (N=28) | p |
| --- | --- | --- | --- | --- | --- | --- |
| Birth characteristics, mean (SD) |  |  |  |  |  |  |
| Birthweight, g | 1089 (203) | **.022** | 2886 (295) | .799 | 3677 (421) | .831 |
| Gestational age, weeks | 28.4 (2.3) | .355 | 39.7 (1.3) | .604 | 39.5 (1.4) | .436 |
| Head circumference, cm (n=15/27/28) | 26.8 (1.8) | .776 | 34.2 (1.0) | .105 | 35.2 (1.2) | .622 |
| Apgar score (n=22/27/28) | 8.7 (1.2) | .491 | 9.5 (1.7) | .106 | 9.9 (0.3) | .671 |
| Male sex, No. (%) | 16 (70) | .200 | 20 (63) | .192 | 11 (39) | .716 |
| WAIS at age 19 years, mean (SD) (n=9/12/11) | 85.6 (19.0) | .565 | 88.4 (11.7) | **.036** | 94.9 (7.5) | .085 |
| Maternal age at birth, years, mean (SD) n=23/28/27) | 26.2 (2.9) | .067 | 29.0 (4.5) | .342 | 29.1 (4.0) | .108 |
| Parental SES, mean (SD) (n=20/13/16) ^a^ | 3.0 (1.1) | .107 | 2.8 (1.5) | **.048** | 3.6 (1.2) | .606 |
| Maternal smoking at conception, No. (%) | - | - | 22/28 (79) | .069 | 13/28 (46) | .549 |

P-values vs participants. Abbreviations: VLBW: Very Low Birth Weight; SGA: Small for Gestational Age; WAIS: Wechsler Adult

Intelligence Scale; SES: Socioeconomic Scale

^a^ SES was collected at 14 years for 18 VLBW, 13 SGA and 14 control non-participants, supplemented at 19 years for 2 VLBW and

2 controls.

**Table S2** Descriptive values of Autism-Spectrum Quotient excluding participants with IQ < 85 at 19 years

|  | **Preterm VLBW** | p | **Term SGA** | p | **Control** |
| --- | --- | --- | --- | --- | --- |
| **AQ**, mean (SD) |  |  |  |  |  |
| Social skill | 1.7 (1.7) | .368 | 1.9 (2.3) | .368 | 1.1 (1.2) |
| Attention switching | 3.8 (2.0) | .249 | 4.4 (2.2) | **.049** | 3.0 (2.0) |
| Attention to detail | 3.2 (2.2) | .500 | 3.6 (2.1) | .500 | 3.1 (2.2) |
| Communication | 2.1 (1.4) | .368 | 2.1 (2.2) | .368 | 1.6 (1.5) |
| Imagination | 2.7 (1.6) | .368 | 2.2 (1.2) | .368 | 2.7 (1.4) |
| Sum score | 13.5 (6.2) | .368 | 13.8 (5.8) | .368 | 11.7 (4.9) |
| **AQ Sum Score ≥20p**, No. (%) | 8/30 (27) | **.035** | 5/38 (13) | .368 | 2/60 (3) |

P-values vs. controls, adjusted according to Benjamini and Hochberg[1].

Abbreviations: VLBW: Very Low Birthweight; SGA: Small for Gestational Age; AQ: Autism-Spectrum Quotient;

n given as: (VLBW/SGA/Control) Sum score(n=30/38/60), Social skills subscale (n=30/39/62),

Attention switching (n=30/39/62), Attention to detail (n=30/40/62), Communication (n=30/39/60),

Imagination (n=30/38/62)

IQ from the 19 years’ follow-up in a subsample of participants at 26 years: n=46/46/68 control,

the number of participants with estimated IQ below 85: n=16/46 (35%) VLBW, 6/46 (13%) SGA

and 5/68 (7%) control

**Table S3** *Confounder analysis*: Linear regression coefficients with ASEBA ASR Total Problems score, AQ sum score and PDI Grand

Total Score as dependent variables in the **preterm VLBW** group vs the control group, adjusted separately for each variable.

|  | **ASEBA ASR** |  | **AQ** |  | **PDI** |  |
| --- | --- | --- | --- | --- | --- | --- |
|  | B (95% CI) | p | B (95% CI) | p | B (95% CI) | p |
| **Sex** |  |  |  |  |  |  |
| Unadjusted | 12.4 (4.9 to 19.9) | .001 | 3.2 (1.2 to 5.2) | .001 | 5.9 (-0.3 to 12.1) | .064 |
| Adjusted | 12.8 (5.3 to 20.3) | .001 | 3.0 (1.0 to 4.9) | .003 | 5.5 (-0.8 to 11.7) | .085 |
| **Parental SES** |  |  |  |  |  |  |
| Unadjusted | 11.7 (3.5 to 19.9) | .005 | 3.8 (1.7 to 5.9) | .001 | 6.0 (-0.6 to 12.5) | .073 |
| Adjusted | 11.8 (3.6 to 20.1) | .005 | 3.8 (1.7 to 6.0) | .001 | 6.0 (-0.6 to 12.7) | .073 |
| **Maternal age at birth** |  |  |  |  |  |  |
| Unadjusted | 12.7 (5.1 to 20.3) | .001 | 3.0 (1.0 to 4.9) | .003 | 5.2 (-1.1 to 11.6) | .107 |
| Adjusted | 12.0 (4.2 to 19.8) | .003 | 3.0 (1.0 to 5.0) | .004 | 5.1 (-1.5 to 11.6) | .128 |

Abbreviations: ASEBA ASR: Achenbach System of Empirically Based Assessment - Adult Self-Report; AQ: Autism-Spectrum Quotient; PDI: Peters et al.

Delusions Inventory; VLBW: Very Low Birth Weight; SES: Socioeconomic Status;

No information on maternal smoking at conception in the preterm VLBW group.

**Table S4** *Confounder analysis*: Linear regression coefficients with ASEBA ASR Total Problems score, AQ sum score and PDI Grand

Total Score as dependent variables in the **term SGA** group vs the control group, adjusted separately for each variable.

|  | **ASEBA ASR** |  | **AQ** |  | **PDI** |  |
| --- | --- | --- | --- | --- | --- | --- |
|  | B (95% CI) | p | B (95% CI) | p | B (95% CI) | P |
| **Sex** |  |  |  |  |  |  |
| Unadjusted | 12.5 (5.2 to 19.7) | .001 | 2.3 (0.4 to 4.2) | .018 | 4.0 (-2.0 to 10.0) | .193 |
| Adjusted | 12.7 (5.4 to 19.9) | .001 | 2.2 (0.3 to 4.0) | .025 | 3.8 (-2.2 to 9.8) | .213 |
| **Parental SES** |  |  |  |  |  |  |
| Unadjusted | 11.3 (3.3 to 19.2) | .006 | 3.0 (1.0 to 5.1) | .004 | 5.8 (-0.6 to 12.1) | .074 |
| Adjusted | 11.3 (3.4 to 19.3) | .006 | 3.1 (1.0 to 5.1) | .005 | 5.8 (-0.6 to 12.1) | .074 |
| **Maternal age at birth**, years |  |  |  |  |  |  |
| Unadjusted | 12.7 (5.0 to 20.4) | .001 | 2.3 (0.3 to 4.3) | .024 | 5.6 (-0.8 to 12.1) | .084 |
| Adjusted | 12.1 (4.3 to 19.9) | .003 | 2.3 (0.3 to 4.3) | .028 | 5.5 (-1.1 to 12.0) | .101 |
| **Maternal smoking at conception** |  |  |  |  |  |  |
| Unadjusted | 12.8 (5.3 to 20.3) | .001 | 2.4 (0.4 to 4.4) | .032 | 6.2 (0.2 to 12.1) | .043 |
| Adjusted | 11.6 (3.9 to 19.2) | .003 | 2.2 (0.2 to 4.2) | .028 | 5.7 (-0.4 to 11.7) | .066 |

Abbreviations: ASEBA ASR: Achenbach System of Empirically Based Assessment - Adult Self-Report; AQ: Autism-Spectrum Quotient;

PDI: Peters et al. Delusions Inventory; SGA: Small for Gestational Weight; SES: Socioeconomic Status

**Table S5** Perinatal risk factors and neurodevelopmental markers in the preterm VLBW group excluding three participants with CP; linear regression with ASEBA ASR Total Problems score, AQ Sum score and PDI Grand Total score separately as dependent variables, and for each variable one at a time as covariate.

|  | **ASEBA ASR** |  | **AQ** |  | **PDI** |  |
| --- | --- | --- | --- | --- | --- | --- |
| Factor (covariate) | β (95% CI) | *p* | β (95% CI) | *p* | β (95% CI) | *p* |
| **Perinatal factors** |  |  |  |  |  |  |
| Gestational Age (weeks n=58/55/57) | -1.7 (-4.1 to 0.7) | .459 | -0.6 (-1.2 to -0.02) | .275 | -1.8 (-4.0 to 0.4) | .224 |
| Birthweight (pr. 100g, n=58/55/57) | -1.6 (-4.1 to 1.0) | .525 | -0.4 (-1.1 to 0.2) | .484 | -2.2 (-4.6 to 0.09) | .165 |
| Days NICU (n=55/53/54) | 0.3 (0.1 to 0.5) ^a^ | .063 | 0.07 (0.02 to 0.12) | .140 | 0.2 (0.06 to 0.4) | .112 |
| Days on respiratory support (n=57/55/56) | 0.9 (0.3 to 1.5) ^b^ | .063 | 0.2 (0.01 to 0.3) | .231 | 0.7 (0.1 to 1.3) | .112 |
| IVH (n=56/54/55) | 0.2 (-8.3 to 8.7) ^c^ | .965 | 1.1 (-0.9 to 3.2) | .556 | 7.8 (0.3 to 15.4) | .165 |
| Maternal glucocorticoid (n=58/55/57) | -2.7 (-15.2 to 9.9) ^d^ | .781 | -0.4 (-3.6 to 2.9) | .905 | -5.9 (-17.5 to 5.7) | .440 |
| Apgar 5 min (n=56/57/55) | -1.7 (-5.3 to 1.9) | .571 | -0.3 (-1.3 to 0.6) | .849 | -1.7 (-5.1 to 1.7) | .440 |
| **Motor function** |  |  |  |  |  |  |
| BSID Psychomotor Developmental Index score at 1 year (n=21/20/21) | 0.02 (-0.6 to 0.6) | .965 | -0.03 (-0.2 to 0.1) | .905 | 0.2 (-0.4 to 0.7) | .594 |
| PDMS Eye/hand coordination at 5 years (n=22/21/22) | -1.4 (-3.2 to 0.3) | .375 | -0.06 (-0.6 to 0.5) | .905 | -1.7 (-3.7 to 0.3) | .224 |
| PDMS Balance at 5 years (n=22/21/22) | -1.5 (-4.6 to 1.7) | .571 | -0.05 (-0.9 to 0.8) | .905 | -2.2 (-5.7 to 1.3) | .364 |
| PDMS Locomotion at 5 years (n=22/21/22) | -0.4 (-1.5 to 0.8) | .720 | -0.1 (-0.4 to 0.2) | .891 | -0.6 (-1.9 to 0.7) | .471 |
| Movement ABC, total score at 14 years (n=39/37/38) | 0.8 (-0.1 to 1.6) | .336 | 0.2 (0.06 to 0.5) | .438 | 0.9 (0.02 to 1.9) | .165 |
| **Cognitive function** |  |  |  |  |  |  |
| WPPSI at 5 years (n=20/19/20) | -0.1 (-0.7 to 0.4) | .751 | -0.01 (-0.2 to 0.2) | .905 | 0.2 (-0.4 to 0.7) | .611 |
| WISC at 14 years (n=41/39/40) ^e^ | -0.1 (-0.4 to 0.2) | .571 | -0.05 (-0.1 to 0.04) | .539 | -0.06 (-0.4 to 0.3) | .711 |

P values adjusted according to Benjamini and Hochberg[1].

Abbreviations: VLBW: Very Low Birth Weight; CP: Cerebral Palsy; ASEBA ASR: Achenbach System of Empirically Based Assessment - Adult Self-Report; AQ: Autism-Spectrum Quotient; PDI: Peters et al. Delusions Inventory; NICU: Neonatal Intensive Care Unit; IVH: Intra Ventricular Hemorrhage; BSID: Bayley Scales of Infant Development; PDMS: Peabody Developmental Motor Scales; WPPSI: Wechsler Preschool and Primary Scale of Intelligence; WISC: Wechsler intelligence scale for children; CPAP: Continuous Positive Airway Pressure

^a^ Information on days in NICU: mean (SD): 66.0 (31.2) - max 160 days

^b^ Information on days on respiratory support (Ventilator or CPAP). (No resp. supp.: n=22 (39%) One day with respiratory support: n=14 (25%) max: 44 days; Among all mean (SD): 4.8 (9.7), among those with respiratory support ≥ 1 day mean (SD): 7.9 (11.4)).

^c^ IVH Grading: (No IVH: 48 (86%), grade 1: n=4, grade 2: n=2, grade 3: n=1, grade 4: n=1)

^d^ Maternal glucocorticoids before birth: (29 no; 29 yes)

^e^ WISC estimated from 4 subtests

**References**

1. Benjamini Y, Hochberg Y: **Controlling the False Discovery Rate: A Practical and Powerful Approach to Multiple Testing**. *Journal of the Royal Statistical Society Series B (Methodological)* 1995, **57**(1):289-300.
